# Introducing a Semi-Coated Model to Investigate Antibacterial Effects of Biocompatible Polymers on Titanium Surfaces

**DOI:** 10.3390/ijms16024327

**Published:** 2015-02-17

**Authors:** Andreas Winkel, Wibke Dempwolf, Eva Gellermann, Magdalena Sluszniak, Sebastian Grade, Wieland Heuer, Michael Eisenburger, Henning Menzel, Meike Stiesch

**Affiliations:** 1 Clinic for Prosthetic Dentistry and Biomedical Materials Science, Hannover Medical School, Carl-Neuberg-Str. 1, D-30625 Hannover, Germany; E-Mails: gellermann.eva@mh-hannover.de (E.G.); grade.sebastian@mh-hannover.de (S.G.); heuer.wieland@mh-hannover.de (W.H.); eisenburger.michael@mh-hannover.de (M.E.); stiesch.meike@mh-hannover.de (M.S.); 2 Institute for Technical Chemistry, Braunschweig University of Technology, Hans-Sommer-Str. 10, D-38104 Braunschweig, Germany; E-Mails: w.dempwolf@tu-bs.de (W.D.); m.sluszniak@web.de (M.S.); h.menzel@tu-bs.de (H.M.)

**Keywords:** antimicrobial surface, polymer coating, bacteria, biofilm, implants, experiments *in vitro*

## Abstract

Peri-implant infections from bacterial biofilms on artificial surfaces are a common threat to all medical implants. They are a handicap for the patient and can lead to implant failure or even life-threatening complications. New implant surfaces have to be developed to reduce biofilm formation and to improve the long-term prognosis of medical implants. The aim of this study was (1) to develop a new method to test the antibacterial efficacy of implant surfaces by direct surface contact and (2) to elucidate whether an innovative antimicrobial copolymer coating of 4-vinyl-*N*-hexylpyridinium bromide and dimethyl(2-methacryloyloxyethyl) phosphonate (VP:DMMEP 30:70) on titanium is able to reduce the attachment of bacteria prevalent in peri-implant infections. With a new *in vitro* model with semi-coated titanium discs, we were able to show a dramatic reduction in the adhesion of various pathogenic bacteria (*Streptococcus sanguinis*, *Escherichia coli*, *Staphylococcus aureus*, *Staphylococcus epidermidis*), completely independently of effects caused by soluble materials. In contrast, soft tissue cells (human gingival or dermis fibroblasts) were less affected by the same coating, despite a moderate reduction in initial adhesion of gingival fibroblasts. These data confirm the hypothesis that VP:DMMEP 30:70 is a promising antibacterial copolymer that may be of use in several clinical applications.

## 1. Introduction

In almost all medical disciplines, implant systems are of increasing importance as a temporary or permanent replacement of lost organ functions. The growing demands of an ever-ageing society for high quality of life and performance—even at great age—are accompanied by accelerated medical progress and increasing acceptance of implant technologies by the patient. Although there have been continuous improvements in implant materials and techniques, as well as tissue integration and compatibility, implant infections still represent a long-term threat to prosthetic treatment in various medical fields, such as orthopedics, dentistry, heart surgery and otolaryngology [1,2]. Implant-associated infections are caused by bacterial biofilms forming on the implant surface. Biofilms are communities with complex structures of bacteria embedded in an extracellular matrix.

Despite all precautions and postoperative procedures, bacterial colonization of implants can never be totally excluded [3,4]. Possible reasons for biofilm formation include intraoperative contamination, systemic spreading and permanent transcutaneous passages. Unfortunately the interaction between implant and tissue—with all its limitations in comparison to a natural interface—supports lasting bacterial adhesion to artificial surfaces, biofilm formation and subsequent infection of peri-implant tissue and, at worst, the loss of the implant or its function. Medical treatment of biofilm-related infections is to this day not effective, since bacteria organized in biofilms exhibit greater resistance to external influences, such as antibiotics and the host’s immune system, than planktonic bacteria [5,6]. If postoperative complications are to be prevented, it would be a major advance if initial attachment and subsequent biofilm formation on implant structures could be prevented with an innovative antibacterial surface.

Previous studies have already shown that the combination of membrane-destroying cationic macromolecules and hydrophobic components interacting with bacteria can cause a significant reduction in bacterial loading on polymer-coated implant material, even though this has only been demonstrated on the basis of single strains [7,8]. Subsequent coating strategies have shown that the adhesion of relevant cells isolated from peri-implant tissue is unaffected. This would increase the possibility that strong bonds can be rapidly formed between the implant and tissue and would make it even more difficult for bacteria to colonize an unsuitable surface in the clinical situation [9,10]. However, these studies were limited by the short bacterial adhesion time (1 h) as well as the selection of only one clinical application. Furthermore, it remains unclear if the observed antibacterial effects are mediated by direct contact between bacteria and surfaces or by release of antibacterial substances from the surface.

The aim of this study was to implement a new experimental method to test the direct contact-based mechanisms of antibacterial effects. Furthermore, the hypothesis was to be tested that the innovative antibacterial copolymer coating based on poly[(4-vinyl-*N*-hexyl pyridinium bromide)-*co*-(dimethyl(2-methacryloyloxyethyl) phosphonate)] (VP:DMMEP 30:70) is effective against a variety of clinically relevant bacterial strains (*E. coli*, *P. aeruginosa*, *S. sanguinis*, *S. mutans*, *S. aureus*, *S. epidermidis*) and at the same time allows sufficient adhesion of human cells.

## 2. Results and Discussion

### 2.1. Implementation of a Semi-Coated Test Model

A well-established approach to determine the biocompatibility or antibacterial activity of a coated substance is to use two different discs per sample run. One disc is coated with the substance and the other is an uncoated disc as control. The limitation of this approach is that—even if there is an antibacterial effect—it remains unclear after microscopy whether this is caused by direct contact or by release of antibacterial substances from the surface coating into the medium. Additional tests would be necessary such as comparison of cfu (colony-forming units) from supernatants or washing solutions to clarify cause and effect. This would include labor-intensive and time-consuming plating and counting processes.

**Figure 1:**
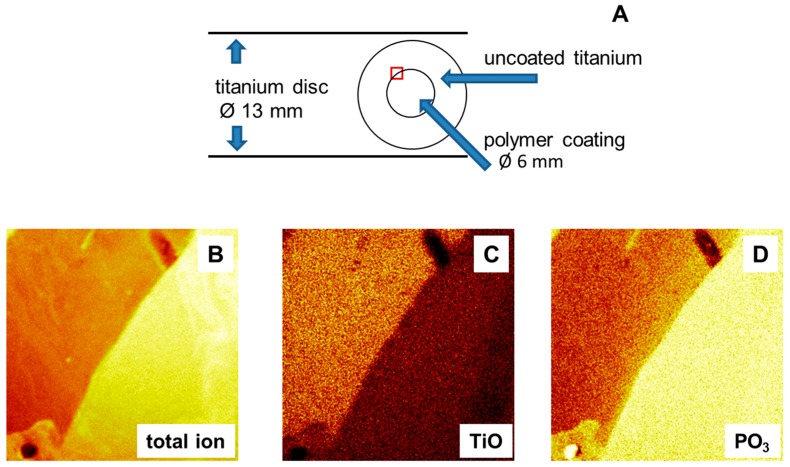
(**A**) Sketch of titanium disc and coating pattern; (**B**–**D**) Secondary ion mass spectrometry images (image size: 500 µm × 500 µm); (**B**) Total ion image at the border between coated (bright yellow) and uncoated areas; and (**C**,**D**) secondary ion mass spectrometry (SIMS) images of the coated area for different ions.

However, if the control experiment and the substance test could be conducted on the same disc, the medium as well as the preparation of bacteria would be the same and the reduction in bacterial adhesion on the coated parts could be directly related to the contact of bacteria with the surface. This is of interest in particular if working with putative microbe-repelling coatings and a broad variety of different surfaces simultaneously. Artifacts due to detached material or soluble substances can be excluded, if there is an unambiguous and defined border between the coated and uncoated areas, as shown in Figure 1B. Moreover, the demonstration of such a border region is the most convincing argument for the target function of an antibacterial coating by just one picture. Thus, the development of an easy and fast partially coating strategy would save material, time and effort regarding preparation and performance of assays in comparison to the conventional approach with completely coated discs if presuming that no artefacts by this new technique occur and for completely coated discs additional microbiological (colony-forming units) or molecular biological analyses would be necessary to address the same mentioned limitation.

For this reason, a new method was designed: Semi-coated titanium discs were prepared with the help of an adhesive foil as template; see Figure 1A. The sample disc was covered with the foil, except 6 mm of the internal diameter, which exposed the pure titanium oxide surface. During the spin coating process, copolymer solution was spread over the whole sample disc. The adhesive foil was removed before tempering, leaving a copolymer film on only the unlaminated part. Sample discs were then heated in an oven at 120 °C for 19 h to finish the binding of the copolymer to the titanium oxide surface. The result were semi-coated titanium discs, with a polymer coated center surrounded by a pure titanium oxide surface, see Figure 1B.

The influence of the foil or the lamination itself was investigated to confirm that the properties of the primary foil laminated part and the pure titanium surface were the same. Contact angle measurements, ellipsometric measurements and tests of antibacterial activity were conducted on titanium surfaces before and after covering with an adhesive foil while passing through the normal coating cycle, e.g., annealing the sample at 120 °C for 19 h.

A layer with a thickness in the range of 2.0 ± 0.1 nm was observed after removing the adhesive foil. Contact angles were in the range of θ_adv_ = 66° ± 2° and θ_rec_ = 45° ± 4°, see Table 1. These results indicated a change in the passivating oxide layer on titanium surfaces. The oxide forms within seconds on the bare metal surface and grows further as a function of time and temperature [11]. Natural oxide layers reported in the literature are in the range of 3–7 nm [12]. Therefore titanium substrates have contact angles of θ_adv_ = 33° and θ_rec_ = 22° directly after polishing and washing. The contact angles increased to θ_adv_ = 84° and θ_rec_ = 64° for aged titanium samples [9]. The observed shift to higher contact angles and the increase in layer thickness after annealing the titanium samples can be explained with a temperature-accelerated aging process, resulting in an increased oxide layer. The adhesive foil itself did not modify the surface properties.

### 2.2. Copolymer Coating

In previous studies, the statistical copolymer (*i.e.*, a copolymer with a statistical sequence of the two monomers) consisting of 4-vinyl-*N*-hexylpyridinium bromide (VP) and dimethyl(2-methacryloyloxyethyl)phosphonate (DMMEP) was intensively investigated with respect to the influence of copolymer composition on the balance between biocompatibility and antimicrobial activity [9,10]. A composition of 0.24 VP and 0.76 DMMEP (VP:DMMEP 30:70) was identified as optimal and chosen as a test substance to compare the new semi-coated sample testing method to the standard method using coated and uncoated samples.

**Table 1:** Coating characteristics of VP:DMMEP 30:70.

Coating Method	Sample	Layer Thickness (nm)	Contact Angle (°)	
			θ_adv_	θ_rec_
semi-coated	VP:DMMEP 30:70	5.0 ± 0.6	68 ± 2	48 ± 6
	titanium area of semi-coated sample	2.0 ± 0.1	66 ± 2	45 ± 4
completely coated	VP:DMMEP 30:70	5.1 ± 1.4	64 ± 3	43 ± 2
uncoated	pure titanium	3–7 ^c^	33 ^a^	22 ^a^
			84 ^b^	64 ^b^

VP: 4-vinyl-*N*-hexylpyridinium bromide; DMMEP: dimethyl(2-methacryloyloxyethyl)phosphonate; ^a^ Directly after polishing and washing [9]; ^b^ Aged titanium sample [9]; ^c^ “Native” oxide film on pure titanium, grown at room temperature [12].

**Figure 2:**
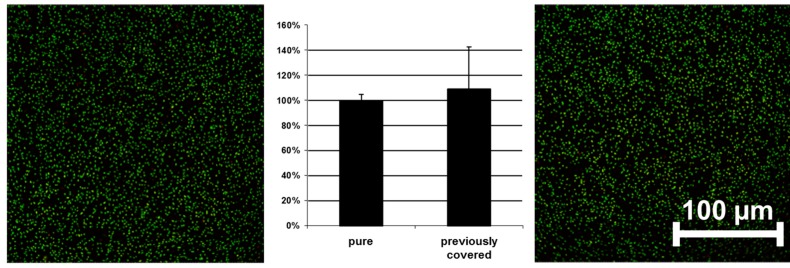
Adhesion of bacteria on pure (**left**) and previously covered surfaces (**right**) without polymer coating (*E. coli* used as example)—confocal laser scanning microscopy (CLSM) pictures and quantified data. Scale bar = 100 μm.

Tests on antibacterial activity were conducted on these surfaces, as shown in Figure 2. No significant difference could be observed between a pure and a previously covered titanium surface. The results of the control experiments clearly indicate that the newly designed semi-coated test model can be used to study the influence of an innovative copolymer coating on different bacteria.

In Table 1, coating characteristics are compiled from different experimental runs, including layer thickness and water contact angle. Comparison of the layer thicknesses of VP:DMMEP 30:70 on completely coated and semi-coated titanium substrates showed that neither coating method affected layer formation. Layer thicknesses of about 5 nm could be obtained, so that no polymer was removed from the surface during detachment of the template. Contact angles varied only slightly and within the error of the measurements, from θ_adv_ 64° ± 3° for complete coating to θ_adv_ 68° ± 2° for semi-coated samples and θ_rec_ 43° ± 2° to θ_rec_ 48° ± 6°, respectively. These specifications provide additional proof for the applicability of the semi-coating method.

In addition, imaging of the chemical surface composition was conducted by secondary ion mass spectrometry (TOF-SIMS); see Figure 1B–D. An image representing the total ion flux of the polymer coated surface is shown in Figure 1B. A distinct border between coated and uncoated areas could be identified, thus confirming the presence of a semi-coated surface. Binding of the copolymer is related to a reaction between the OH-groups of the metal oxide surface and the phosphonate groups of the DMMEP component. The phosphorous-containing ions can therefore be taken as an indicator of polymer coating (Figure 1D). This was further confirmed using the CN ions (data not shown), as nitrogen atoms are present in the copolymer in the VP fraction. TOF-SIMS measurements are highly surface sensitive, so that the signal intensity of titanium ions detected beneath the coating was lower than in the uncoated part (Figure 1C).

### 2.3. Antibacterial Activity

**Figure 3:**
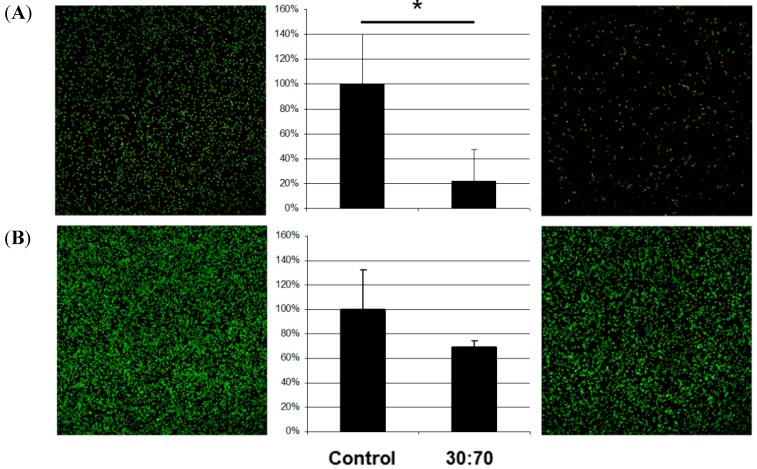

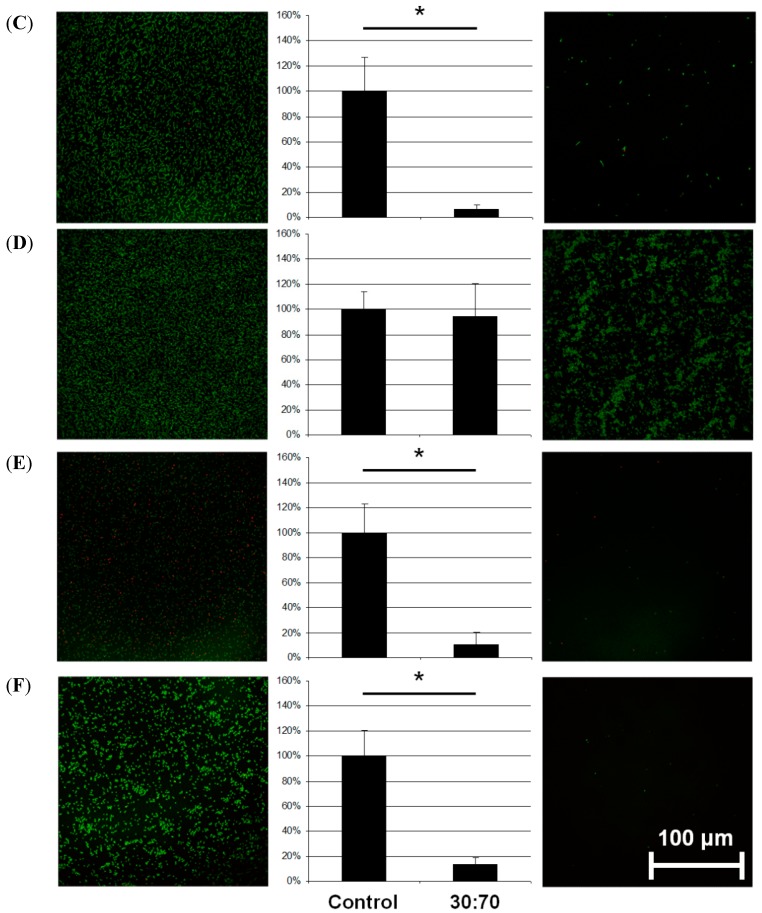
Adhesion of different bacterial species (**A** = *E. coli*; **B** = *P. aeruginosa*; **C** = *S. sanguinis*; **D** = *S. mutans*; **E** = *S. aureus*; **F** = *S. epidermidis*) on titanium discs coated with VP:DMMEP 30:70 (**right**) relative to the uncoated control (**left**)—CLSM pictures and quantified data (* *p* < 0.05). Scale bar = 100 μm.

The effect on bacteria by the coating depended on the species (Figure 3). *S. aureus* and *S. epidermidis* are members of the natural skin flora. When displaced inside the body they can nevertheless cause serious infections, especially in enervated or immune deficient persons; they are common in peri-implant infections after orthopedic or trauma surgery [13]. In this study, coating with VP:DMMEP 30:70 led to a 90% decrease in the bacterial load of *S. aureus* and *S. epidermidis* after 5 h of incubation in comparison to uncoated samples. This confirmed results from a previous study with only 1 h incubation [14]. Similar or even better results could be achieved for *S. sanguinis*, a primary colonizer of human teeth and dental implants. Therefore, coating dental or orthopedic implants with VP:DMMEP is an option in the future worth additional study.

Regarding the adhesion of *E. coli*, a typical pathogen in smear infections, the coating was slightly less effective than in the cases of *S. aureus*, *S. epidermidis* or *S. sanguinis*, but still exhibited distinct antibacterial activity. In comparison with streptococci and staphylococci, the gram-negative *E. coli* bacteria are mobile and less influenced by changes in adhesion. The same argument holds for *P. aeruginosa*, which forms biofilms on lens tubes (in cases of long-term artificial respiration) and devices in otolaryngology causing serious infections in these areas. However, the antibacterial effect for this species was less than 40% and not significant. Accordingly, a coating of devices in otolaryngology or on lens tubes might be less effective.

In contrast to previous results with *S. mutans* [9] the present experiments showed no clear reduction in bacterial adhesion. However, the bacteria did not adhere homogenously to the surface, but aggregated to some extent. Such aggregation has been interpreted as a stress reaction after bacteria are placed on an unfavorable surface [15] and has been observed for other combinations of VP:DMMEP copolymers with higher VP proportions, as well as with other bacterial species [9,10]. In the current study we incubated the bacteria for 5 h, in contrast to 1 h in previous studies. The results indicate that *S. mutans* was initially barely able to adhere to the coating, but grows nevertheless, although it is stressed by the surface.

Our approach aimed to the initial healing process directly after implant insertion, when bacteria and periimplant host tissue compete in adhesion to the implant surface. In this period a significant reduction of bacterial colonization might give cells the advantage in this “race to the surface” to establish a stable wound closure that prevents biofilm progression. Since the adhesion of some bacterial species is reduced, but the proportion of dead bacteria (detected by LIVE/DEAD staining) remains stable in this study (data not shown), VP:DMMEP 30:70 coatings could be regarded as repellent to bacteria [16]. In recent decades, several coating designs for implant surfaces have been described that employ this approach [17,18,19,20]. However, most of these studies have focused on only one or two bacterial species. In the light of the wide range of the diversity and characteristics of bacteria in different biofilms, this limits their application *in vivo*.

Other approaches used releasing mechanisms, provided killing on contact [21,22,23], or a combination of both [24,25]. One unsolved problem is the depletion of the established pool of the antibacterial substance over time, along with the increasing risk of creating resistances within the bacterial species. Another problem is the gradually masking of a bactericide surface by the remaining dead bacteria, which might serve as a safe substrate for subsequent bacteria. However, the mode of action of a new coating and its usefulness and risks can only be reliably assessed when there is unambiguous evidence as to whether the effect is generated by killing on contact or by the release of the coating material.

**Figure 4:**
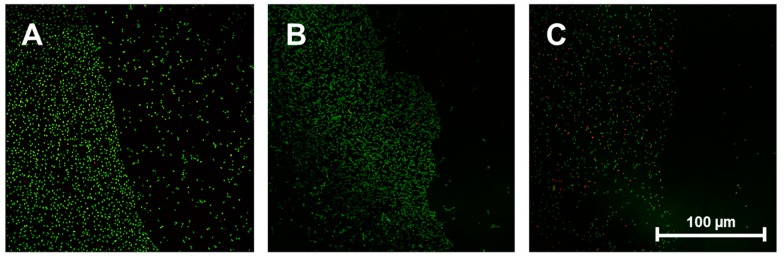
Border region between uncoated (left side of each picture) and coated areas (right side of each picture) of titanium discs after seeding with *E. coli* (**A**), *S. sanguinis* (**B**) and *S. aureus* (**C**). Scale bar = 100 μm.

In order to address the question of whether the observed antibacterial effects are caused just by contact or by soluble fragments of the coating or residues of the coating/washing processes, additional pictures of the border regions on semi-coated titanium discs were taken. Figure 4A–C show typical results for *E. coli*, *S. sanguinis* and *S. aureus* (which displayed markedly reduced adhesion on VP:DMMEP 30:70). These exhibit a defined border line between coated and uncoated areas of the discs, as bacterial colonization is potently reduced on the polymer coating. This experiment clearly demonstrated that the reported antibacterial effects are related to the direct contact between bacteria and the modified surface, whereas nearby areas free of VP:DMMEP 30:70 showed normal bacterial adhesion, without any signs of putative antibacterial residues or components dissolved during culturing. This confirms the first part of the hypothesis—that the innovative antibacterial copolymer coating is effective against a variety of clinically relevant bacterial strains.

### 2.4. Adhesion, Proliferation, and Morphology of Human Fibroblasts

Thus, this titanium surface modification demonstrated a dramatic antibacterial effect for several bacterial species, but might not be able to prevent microbial attachment completely, as has been reported for various polymer coatings [18,21,25,26]. It is therefore even more important that for certain applications in implant coating (like prosthetic dentistry or orthopedics) the polymer supports or at least permits strong tissue binding to suppress subsequent bacterial growth. We showed in previous studies that some polymer coatings cause a crucial decrease in adhered cells [9]. It is important to investigate antibacterial effects at a very early stage as well as cytocompatibility, an aspect often overlooked.

Adhesion, proliferation and morphological changes in both gingival and dermis fibroblasts were tested *in vitro*, in order to verify the compatibility between VP:DMMEP 30:70 and relevant soft tissues. The number of attached cells was calculated from overall lactate dehydrogenase (LDH) activity. Microscopic evaluation was carried out after different incubation times. In all cases non-coated titanium discs served as a reference and related cell numbers were set at 100%.

**Figure 5:**
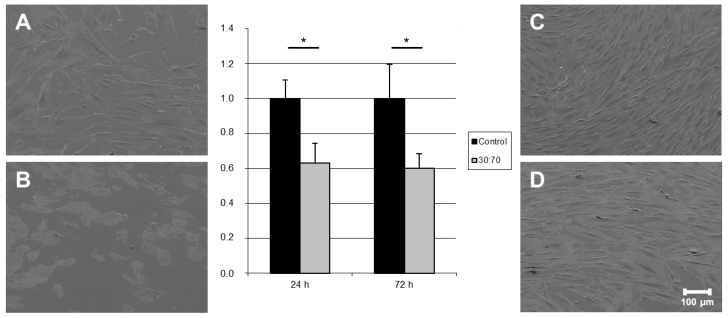
Quantification and visualization (SEM) of adhered human gingival fibroblasts on titanium discs coated with VP:DMMEP 30:70 (**B** = 24 h; **D** = 72 h) in relation to the uncoated control (**A** = 24 h; **C** = 72 h)—SEM pictures and quantified data (* *p* < 0.05). Scale bar = 100 μm.

Gingival fibroblasts (HGFIBs) are the most important type of soft tissues surrounding dental implants and are crucial for effective wound closure in an area with constant high bacterial load [27]. The antibacterial polymer coating VP:DMMEP 30:70 resulted in a reduced initial adhesion of HGFIB to the surface. This was reflected in the significant reduction of adherent cells by 40% compared to the control value (Figure 5). Such a reduction could arise from an unsuitable chemical composition or topography of the surface, changes in wettability or surface co*n*taminations depending on the used cell type [28]. Since topography and water contact angle varied only slightly, the inefficient focal adhesion of HGFIBs on the specific chemical composition of the statistical copolymer might be responsible for this effect. This would match also to the higher proportion of round fibroblastic cells in comparison to the uncoated control visualized in scanning electron microscope (SEM) after 24 h (Figure 5A,B). However, these effects seem to apply just to the adhesion process itself. Once completely adhered, gingival fibroblast behaved adequately on both surfaces, with normal fibroblast morphology and comparable growth rates (Figure 5C,D). No signs of cell toxicity in terms of a reduction in proliferation were detected, as these would have caused a significantly lowered cell number (in %) after 72 h. Instead we got a reduction of about 40% in relation to the uncoated titanium at both time-points without any significant difference indicating that on both surfaces (coated with VP:DMMEP 30:70 or uncoated titanium) the proliferation rate within 48 h is nearly the same. A detailed analysis of the reasons for the reduced initial adhesion despite an unaffected proliferation and viability was beyond the scope of the presented study but should be addressed in further investigations.

**Figure 6:**
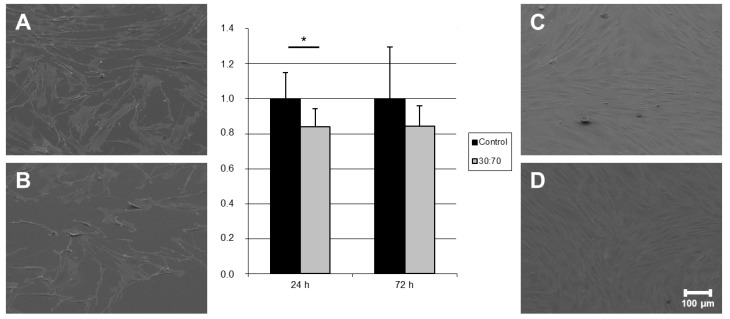
Quantification and visualization (SEM) of adhered human dermis fibroblasts on titanium discs coated with VP:DMMEP 30:70 (**B** = 24 h; **D** = 72 h) in relation to the uncoated control (**A** = 24 h; **C** = 72 h)—SEM pictures and quantified data (* *p* < 0.05). Scale bar = 100 μm.

Adhesion of human dermis fibroblasts (HDFIBs) is an essential part of skin- and wound-related healing processes and is important for all transcutaneous prostheses, catheters or similar devices. Fibroblasts from dermis showed only a slight but in case of 24 h still significant reduction in adhered cell numbers on coated samples—in contrast to HGFIBs—with no morphological abnormalities (Figure 6). Such a different behavior of divers cell types on the same surface was described already for topographies [29] and chemical binding sites [30] demonstrating specific dependence on a suitable distribution of adhesion points and composition of chemical receptors. The decrease of about 20% appeared stable between the first and third days of incubation. This observation indicates again a comparable proliferation rate of cells on uncoated material and was supported by SEM images.

In conclusion, coating titanium surfaces with the copolymer VP:DMMEP 30:70 has no negative impact on biocompatibility, as assessed by the proliferation and viability of cells isolated from peri-implant soft tissue, although initial adhesion might be affected. Therefore, the second part of the hypothesis—that the polymer coating exhibits sufficient adhesion of human cells—is confirmed. However, especially regarding human gingival fibroblasts further optimization of the copolymer composition should have the objective of improving initial cell adhesion onto the coating, as this would accelerate attachment of sealing tissue. The fast and severe adhesion of connective tissue remains one of the major differences between implants and native structures, which prevents epithelium downgrowth and bacteria proceeding [28].

## 3. Experimental Section

### 3.1. Polymer Synthesis

Monomer synthesis of dimethyl(2-methacryloyloxyethyl)phosphonate DMMEP was carried out according to Bressy-Brondino *et al.* [31]. The monomer 4-vinylpyridine (VP) was purchased from Acros Organics (Geel, Belgium) and was dried by distillation from CaH_2_ prior to use. Free radical copolymerization of DMMEP with VP and N-alkylation with 1-bromohexane was carried out as described by Pfaffenroth *et al.* [9].

### 3.2. Preparation of Titanium Surfaces

Ti6Al4V (grade 5) discs of 13 mm in diameter were provided by Otto Bock Healthcare (Duderstadt, Germany). The discs were ground with 800, 1200 and 2500 grit silicon carbide paper and polished with colloidal silica (type MasterMet and MasterMet 2, Buehler GmbH, Düsseldorf, Germany) on a ChemoMet Polishing Cloth from Buehler (Esslingen, Germany). The discs were then each rinsed twice in dichloromethane, acetone, methanol and Millipore water under sonication for 10 min, dried in a stream of nitrogen and stored under vacuum at 120 °C. The substrates were sonicated in HPLC-grade solvents before use: twice in dichloromethane, acetone, methanol and twice in Millipore water for 10 min each. They were dried in a stream of nitrogen for further experimental use.

### 3.3. Surface Coating and Characterization

Semi-coated titanium discs were prepared with the support of a template. An adhesive foil was used, which was cut according to the diameter of the samples (13 mm). An opening of 6 mm in diameter was stamped in the middle of this foil. Titanium discs were pasted up with this template. A solution of the polymer (10 mg/mL) was spin coated onto the titanium discs. The template was then removed and the discs were heated in an oven at 120 °C for 19 h. Then the discs each were sonicated six times in HPLC-grade methanol for 10 min to remove unbound polymer. Complete coating of the substrates was achieved by following the same procedure without a template.

Polymer film thickness was determined using a Multiskop (Optrel, Berlin, Germany) in the null ellipsometer mode. Each titanium disc was measured prior to the polymer coating process as reference. Five ellipsometric measurements were made at different spots for each sample. For semi-coated substrates, three measurements were conducted at the outer uncoated part (titanium oxide surface) and two at the polymer coated middle part. Polymer layer thicknesses were calculated on the basis of a single film model.

After cleaning the polymer coated surfaces, water contact angles were measured on a G1 (Krüss, Hamburg, Germany), using the tilting plate method with a tilt angle of 45°. Five measurements at different positions were recorded for each substrate. In the case of semi-coated substrates, three measurements were conducted at the outer uncoated part (titanium oxide surface) and two at the polymer coated middle section.

TOF-SIMS spectra were acquired by a Physical Electronics PHI 7200 spectrometer (Chanhassen, MN, USA). An 8 keV Cs^+^ primary ion source in pulsed mode was used. Spectra were recorded for positive and negative secondary ions. Positive ion spectra were calibrated using the CH_3_^+^, C_2_H_3_^+^ and C_3_H_5_^+^ peaks, and negative ion spectra were calibrated using the CH^−^, C_2_H^−^ and OH^−^ peaks.

### 3.4. Antibacterial Testing

Semi-coated titanium was tested for differences in bacterial adhesion using various species with clinical relevance for implant infections (*E. coli*—DSM 1103; *P. aeruginosa*—ATCC BAA-47; *S. sanguinis*—DSM 20567; *S. mutans*—DSM 20523; *S. aureus*—DSM 20231; *S. epidermidis*—DSM 20044). All bacteria were cultured overnight, washed twice and finally resuspended after mixing thoroughly in 50 mM Tris HCl buffer (pH 7.5). To ensure equal spreading, the bacterial suspensions were adjusted to final values of 8 × 10^7^ cfu/mL (*E. coli*), 1 × 10^7^ cfu/mL (*P. aeruginosa*), 5 × 10^7^ cfu/mL (*S. sanguinis*), 1 × 10^8^ cfu/mL (*S. mutans*), 1 × 10^9^ cfu/mL (*S. aureus*) or 6 × 10^6^ cfu/mL (*S. epidermidis*) according to optical density measurements at 600 nm. The bacteria were allowed to adhere onto the sample surfaces by incubation in a wet chamber under gentle rotation for 5 h at 37 °C (*E. coli* and *P. aeruginosa* were seeded without rotation but statically, *P. aeruginosa* at room temperature). Discs were then rinsed several times with PBS (L1825; Biochrom AG, Berlin, Germany) to remove unattached cells. Adhered bacteria were stained with LIVE/DEAD BacLight Bacterial Viability Kit (L7012; Invitrogen, Carlsbad, CA, USA), fixed with 2.5% glutaraldehyde (3778; Roth, Karlsruhe, Germany), and visualized by confocal laser scanning microscopy (CLSM; Leica Upright, Heidelberg, Germany) at 63-fold magnification.

Resulting pictures for both emission spectra (495 to 540 nm for SYTO 9; 595 to 750 nm for propidium iodide) were merged. Bacteria were distinguished because of the mixed color in a living (green) and dead (red) subpopulation and quantified separately using WCIF ImageJ (National Institutes of Health, Bethesda, MD, USA).

### 3.5. Adhesion and Proliferation of Human Cells

Human gingival fibroblasts (HGFIB, Cat. No.: 121 0412) and human dermis fibroblasts (HDFIB, Cat. No.: 121 0411) were purchased from Provitro GmbH (Berlin, Germany) and used for biocompatibility testing. Cells were cultured in Dulbecco’s modified Eagle’s medium (FG0435; Biochrom AG) supplemented with 10% fetal bovine serum (P270521, PAN-Biotech GmbH, Aidenbach, Germany), 100 U/mL penicillin and 100 μg/mL streptomycin (A2212; Biochrom AG). Incubation took place at 37 °C in a 5% CO_2_, 95% humidified air atmosphere.

As described previously, a modified LDH activity assay was used to evaluate the quantity and viability of cells on polymer coatings in comparison to uncoated titanium [9,10,32]. In summary, completely coated in comparison to uncoated discs were covered with cell suspensions at a density of 1.5 × 10^4^ cells/mL. Samples were washed in HBSS (L2035, Biochrom AG) after an incubation period of 24 or 72 h to remove unattached cells and media residues. Adhered fibroblasts were lysed using 10% Triton X-100 (93416; Sigma-Aldrich Chemie GmbH, Steinheim, Germany) and incubated at 37 °C for 30 min. Released lactate dehydrogenase was quantified by staining solution (Cytotoxicity Detection Kit, 11 644 793 001, Roche Diagnostics GmbH, Mannheim, Germany) and cell numbers were calculated by comparison with a corresponding standard curve.

The cell morphology of the fibroblasts on surface was analyzed by SEM. Samples were rinsed with PBS (L1825; Biochrom AG) and fixed for 2 h in 2.5% glutaraldehyde diluted in 0.1 M cacodylate buffer. Samples were dehydrated in graded ethanol solutions before applying critical point drying. SEM samples were mounted on stubs and sputter coated (POLARON Sputter Coater SC7500, Quorum Technologies, Newhaven, UK) with a thin layer of gold and examined in an SEM 505 (Philips GmbH, Hamburg, Germany) at 5 kV.

### 3.6. Statistical Analysis

All statistical analyses were performed with the data processing program IBM SPSS Statistics Version 22 for Windows (SPSS, Inc., Chicago, IL, USA). Non-parametric Mann-Whitney-*U*-Tests were performed to compare biological effects on different surfaces. A *p*-value lower 0.05 was considered statistically significant.

## 4. Conclusions

The design and development of a semi-coated test model makes it possible to investigate *in vitro* even slight differences in the effects of polymer coatings on initial bacterial adhesion and growth on implant surfaces. The differences are easily measurable, as a result of a sharp border between coated and uncoated areas. The model excludes sample to sample variations between coated and non-coated samples. Therefore, the effect observed can be ascribed to direct surface contact and clearly distinguished from effects caused by detached material or soluble substances. We see a major benefit of this method using easy-to-prepare semi-coated discs in the fast comparable screening of a big variety of different microbe-repelling polymer compositions and coatings regarding changes in LIVE/DEAD proportion and the limitation of effects to contact induction by microscopy only without the need for further microbiological techniques.

The polymer coating VP:DMMEP 30:70 proved to be effective *in vitro* against bacterial species relevant in different medical disciplines. After an expanded incubation time of 5 h, the observed reduction remains stable in most cases (excepted *S. mutans*), even if single pathogens (*P. aeruginosa*) seem to be less affected by the surface in general. At the same time, these modified surfaces cause small (HDFIBs) or moderate (HGFIBs) decrease in initial cell adhesion. The viability and growth of human fibroblasts from peri-implant soft tissue seem not to be impaired at all. Consequently, the investigated polymer is a promising candidate for an application as antimicrobial coating for a wide variety of medical implants, especially for prosthetic systems with permanent transcutaneous passage. However, the findings made *in vitro* can never be fully extrapolated to the clinical situation and therefore might serve as a valuable indicator but still have to be validated in preclinical animal models.
